# Children visiting the ICU

**DOI:** 10.1186/cc9945

**Published:** 2011-03-11

**Authors:** KK Borges, L Genaro, M Monteiro

**Affiliations:** 1 Clinica Sao Vicente, Rio de Janeiro, Brazil

## Introduction

Most hospitals only allow children above 12 years old to visit adult ICU patients. However, younger children participating in the hospitalization process manifest, through their family members, their willingness to visit their hospitalized relatives. This raises different healthcare team members' opinions on how to manage their visits to the ICU and prevent psychological harm. This study suggests some relevant steps to allow and receive a child in an adult ICU.

## Methods

A literature review on children visiting the ICU was performed to construct the steps. The flowchart was based on Torres' studies of the child in the face of death, based on Piaget's cognitive development. The flowchart: Identify the family request, either to the psychologist or to the team, to allow the child's visit/Understand the family context and information provided to the child/Healthcare team discussion - team consensus and ICU routine adjustment/Psychologist interview and accompaniment to the bed/After-visit evaluation and follow-up during the ICU stay from family information.

## Results

The literature search has shown diversified results. The use of the flowchart, adjusted to each case requirement, has been very useful in our institution's practice. We could perceive that the healthcare team feels more serene and confident with this guidance and that the families feel more relieved and assured by sharing their afflictions related to their children.

## Conclusions

The interdisciplinary work is fundamental to using the flowchart, requiring a healthcare team aligned with its aims and, above all, sensitive to the essence of the bioethical principle of autonomy, ruled by the patient's and family member's will. However, due to this subject relevance and sensitivity, new discussions are required to deepen the studies and therefore systematize children's visits to ICUs.
